# Complete Genome Sequence of BK Polyomavirus Subtype Ib-1 Detected in a Kidney Transplant Patient with BK Viremia Using Shotgun Sequencing

**DOI:** 10.1128/genomeA.01474-16

**Published:** 2017-02-09

**Authors:** Ravi Ranjan, Asha Rani, Daniel C. Brennan, Patricia W. Finn, David L. Perkins

**Affiliations:** a Department of Medicine, University of Illinois, Chicago, Illinois, USA; b Division of Renal Diseases, Washington University School of Medicine, St. Louis, Missouri, USA; c Department of Microbiology and Immunology, University of Illinois, Chicago, Illinois, USA; d Division of Nephrology, University of Illinois, Chicago, Illinois, USA; e Department of Surgery, University of Illinois, Chicago, Illinois, USA; f Department of Bioengineering, University of Illinois, Chicago, Illinois, USA

## Abstract

We report here the complete genome sequence of polyomavirus BK subtype Ib-1, isolate AR11, identified in urine from a human kidney transplant recipient with a clinical diagnosis of BK viremia. The AR11 isolate is closely related to reference strain human polyomavirus 1 isolate J2B-2 with 99% identity.

## GENOME ANNOUNCEMENT

BK polyomaviruses are members of the family *Polyomaviridae* and are nonenveloped double-stranded DNA viruses that can cause clinical disease in immunocompromised patients (1–3). BK virus-associated nephropathy (BKVN) develops in approximately 10% of kidney transplant patients, resulting in kidney dysfunction and graft failure (3, 4). There are four main BKV subtypes (I to IV) based on genome sequences, with subtype I further divided into four subgroups (Ia, Ib-1, Ib-2, and Ic), which may have different virulence potentials (1). BKV is seroprevalent, and it is unclear whether a particular subtype or a variant plays a role in the pathogenesis of BKVN. The specific nucleotide substitutions can alter amino acid residues in core proteins and may play a critical role in the pathogenicity, specificity, and viability of the viruses (5). There are no specifically approved therapies for the treatment of BKVN; however, a reduction in immunosuppression has been successful for the control of BKVN. However, a reduction in immunosuppression can increase the risk of acute graft rejection in many patients (6, 7). Here, we report the complete genome sequence of polyomavirus BK subtype Ib-1 isolate AR11, identified from the urine from a kidney transplant patient with BK viremia.

A urine sample was collected from a kidney transplant patient, and virus-like particles were pelleted by ultracentrifugation. DNA was isolated, and a library was prepared using the NEBNext DNA library prep kit (New England BioLabs) for sequencing on Illumina HiSeq 2000 with a 50-read-length single-end sequencing chemistry (BGI, Americas). The raw sequence reads were trimmed, quality filtered, and *de novo* assembled to obtain a complete genome sequence using CLC Genomics Workbench (Qiagen, USA). The assembly resulted in a contig of 5,141 bp (total, 7,732,040 sequencing reads), with an average coverage of 75,698× with 100% coverage of the genome. The BLAST analysis of the contig revealed identification of a complete genome sequence and matched to the reference genome sequence of human polyomavirus 1 (GenBank accession no. AB301099). The complete genome exhibits the typical organization of polyomavirus genome, encoding VP1, VP2, VP3, agnoprotein, and small T- and large T-antigen proteins. More than 100 polyomavirus BK strains have been reported worldwide, and these strains have revealed a highly conserved genome with subtype-specific genomic variations.

The alignment of genome sequence of AR11 with reference genome (GenBank accession no. AB301099) revealed 99% identity and query coverage, with a total of six mismatches with three gaps. In the genome at nucleotide level, we detected a substitution of a cytosine for an adenine (at position 203). We also detected multiple substitutions in the genome (positions 2737 to 2757), resulting in mutations in the large T-antigen protein (positions 645 to 651). No substitutions were detected in other major viral proteins, suggesting that the isolate encodes proteins identical to those in the reference strain. Phylogenetic analysis was conducted using the maximum likelihood method based on the Tamura-Nei model, with 1,000 bootstraps, to 81 complete BKV genomes using MEGA5 (8). Isolate AR11 grouped most closely with genotype Ib-1 isolates J2B-2 and A-68H (NCBI nucleotide GenBank accession numbers AB301099 and AB369094, respectively), which were sequenced from urine samples.

### Accession number(s).

The genome sequence of isolate AR11 has been deposited in GenBank under the accession no. KY132094.
